# Effect of Stretching on Thermal Behaviour of Electro-Conductive Weft-Knitted Composite Fabrics

**DOI:** 10.3390/polym14020249

**Published:** 2022-01-08

**Authors:** Md. Reazuddin Repon, Ginta Laureckiene, Daiva Mikucioniene

**Affiliations:** Department of Production Engineering, Faculty of Mechanical Engineering and Design, Kaunas University of Technology, LT-51424 Kaunas, Lithuania; ginta.laureckiene@ktu.lt (G.L.); daiva.mikucioniene@ktu.lt (D.M.)

**Keywords:** compression, conductive textiles, heat generation, knitted structure, stretching

## Abstract

This experiment presents a study carried out on the electric charge passing textiles for heat production in compression weft-knitted composite fabrics used for medical purposes. The aim was to flourish compression support of knitted structure with integrated highly sensitive metal (silver) coated polyamide multifilament yarns and to evaluate its heat origination attributes after stretching in different levels as well as changes of the temperature during the time. A flat double needle-bed knitting machine was utilized to fabricate the selected specimens together with elastomeric inlay-yarn incorporated into the structure for compression generation and silver coated polyamide yarn laid as ground yarn in a plated structure for heat generation. Six different variants depending on the metal coated yarn amount used and the fabric structure along with two types of the conductive yarn linear density were fabricated for this research work. Scanning electron microscope (SEM) images were preoccupied to show the morphology of conductive yarn and thermal pictures were captured to study the evenness of the heat over the surface of composite fabrics depending on conductive yarn distribution in the pattern repeat. The temperature profile of fabricated composite fabrics and comparison of the heat generation by specimens after stretching in different levels was studied

## 1. Introduction

Smart clothing made with conductive materials is required to pass electrical current through the fabric [1]. Heated textiles can be produced by producing a textile fabric first and then integrating electronic components or by producing an electro-conductive yarn with electronic features and then producing a textile of that yarn [2,3,4]. The most important function of heating fabric is to control the temperature generated on the conductive material. Several kinds of textile products such as woven, knitted, and nonwoven fabric, and embroidery can be engaged for assembling textile heating elements [5,6,7]. Warming components made of nonwoven textiles have ended up being of little use, attributable to less electrical conductivity. Due to structural geometry, the resistance of warming components made of woven textile is lower than that of knitted items, despite having identical size and dimensions. Heating textiles have a wide range of end-uses, including medical applications like orthopaedic supports, electrotherapy, clinical cover for keeping up patient’s internal heat level, strain sensors, movement tracking gadgets, etc.; engineering or technical objectives such as motorbike gloves, household use like heating pads, leisure and sport wears, etc. [8,9,10].

There are many research works dedicated to the construction, properties and behaviour of knitted compression structures used for orthopaedic supports [11,12,13,14]. The majority of orthopaedic compressions are prepared, followed by combined structure along with elastomeric inlay-yarns. Furthermore, any additional stiff parts employed in orthopaedic supports for medical and wear comfort functions are recognized to have a considerable impact on the compression caused by the support [14]. It creates additional difficulties in the stage of designing, as all non-stretchable elements have to be taken into account when evaluating compression levels. There are not many investigations published in the field of electro-conductive knitted fabrics used for heat generation of orthopaedic compression supports.

Heat therapy actively works to decrease stiffness in the joints and reduce pain. Heat mitigates the body pain by governing the emergence of pain metabolites as well as ensuring improved blood flow, favourable muscles and connective tissue pliability, joint flexibility and stiffness, etc. [7,15,16]. The temperature for knee rehabilitation should be around 40–45 °C [17]. It signifies that this temperature must be produced and sustained for a specific period of time. Furthermore, to minimize fluctuations in support compression, the heating of the orthopaedic support must be addressed without the use of extra hard elements. However, there are few studies on heated compression supports for orthopaedic treatment that have been reported in the literature.

Compression garments are allocated knitted orthopaedic supports, and the amount of compression is regulated by the garment size including the fabric’s subsequent expansion. To create extreme stretchability and adequate compression, the compression garment fabric is usually made with a stretchable structure and incorporates elastomeric yarns [13,18]. Orthopaedic support compression criteria are not yet standardized. However, four compression classes are recognized by the German Standard RAL–GZ–387/1:2008 based on the employed model of knitted orthopaedic supports; light compression class: 1 (18–21 mmHg) or 2 (23–32 mmHg), strong compression class: 3 (34–46 mmHg) or 4 (>49 mmHg) [11,19].

The stretching way textile materials when subjected to applied stresses have a massive impact on their handling and ultimate use. The stretchability of knitted materials has been studied extensively [19,20,21]. However, there are deficiencies in research on heated compression supports used for orthopaedic therapy during stretching that have been issued in the writing.

Therefore, the purpose of this exploration was to construct a knitted structure for compression support with integrated metal (silver) coated polyamide yarns and to investigate stretching effect of composite fabric on heat generation attributes. The temperature fluctuations during the time and applied voltage function were also examined to measure the feasibility. The impact of Ag-PA yarn linear density, amount of conductive yarn utilized and distribution of metal coated yarn in the knitting pattern on a heating profile were likewise evaluated.

## 2. Materials and Methods

**Figure 1:**
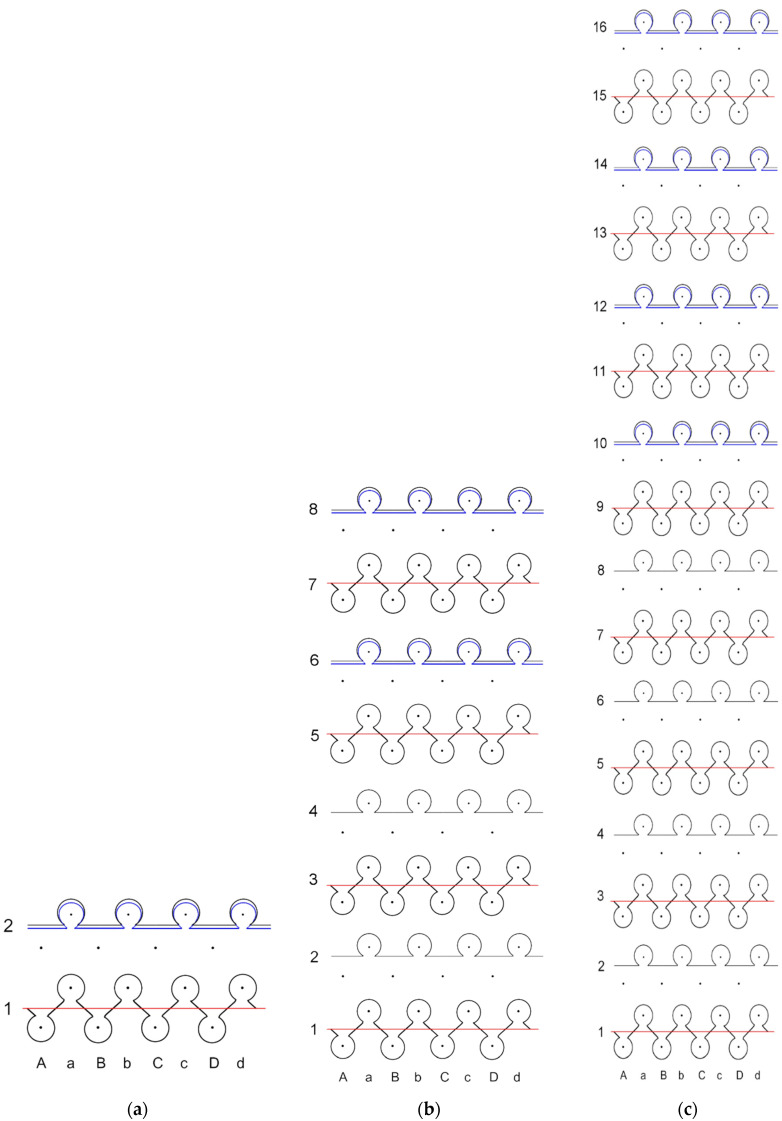
Principle scheme of conductive yarn layout and sample identification: (**a**) CF_LY1 and CF_HY1; (**b**) CF_LY2 and CF_HY2; and (**c**) CF_LY3 and CF_HY3. (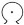 PA6.6 ground yarn, 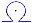 PA + Ag conductive yarn (66 tex for CF_LY and 235 tex for CF_HY), 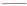 Elastomeric inlay yarn).

The ELITEX^®^ skin contact (SC) silver coated polyamide multifilament yarns of 66 tex/12 filaments and 235 tex/34 filaments were incurred from Imbut GmbH (Greiz, Germany). A flat double needle-bed 14E gauge knitting apparatus (CMS 340TC-L, STOLL, Germany) was employed to fabricate the samples in a combined half-Milano rib structure with elastomeric inlay-yarn incorporated into the structure. The half-Milano rib formation was preferred with the intention of positioning metal coated yarns on one surface; hence, charge transferring yarns were only employed in the single jersey courses. Furthermore, the metal coated yarns were plated by the PA6.6 yarn to protect the structure. The arrangement of 7.8 tex × 4 PA6.6 and 4.4 tex PU (polyurethane) double covered by 4.4 tex PA6.6, yarns were employed as the ground yarn for construction of the rib courses. Metal coated PA yarns were used in plated single jersey courses as ground yarn, while 7.8 tex × 4 PA6.6 yarn was used as plated yarn in these single jersey courses. For elastomeric inlay yarn, 114.5 tex PU, double covered by 7.8 tex × 4 PA6.6, were used and laid in each second course. Three unique samples of each variant (total six) were developed depending on the Ag coated yarn amount and distribution in the composite knit structure along with two categories of conductive yarn linear density and compared the heat generation profile. The detailed principle scheme of metal coated yarn layout and sample identifications of the designed composite fabric used in this study is mentioned in Figure 1.

Essential parameters of designed composite fabrics are presented in Table 1. The areal density of the samples was determined using a GSM cutter (James Heal, Halifax, UK) and a weighing balance (KERN EW 150–3 M, Burladingen, Germany). The wale and course density were measured with a counting glass according to standard LST EN 14971:2006, and the loop length was measured with a HATRA Course Length Tester (SDL Atlas, Rock Hill, SC, USA). The generated temperature and resistance were measured by IR thermometer and Signstek Multimeter. The high resolution field emission scanning electron microscope (FEI Quanta 200 FEG, FEI company, Hillsboro, OR, USA) was used to capture SEM images and a thermal imager (FLIR DM285, FLIR Systems, Wilsonville, OR, USA) was used for thermal imaging of the specimens. Knitted composite fabrics were stretched to course direction at different levels, i.e., 10%, 15% and 20% using an Instron tensile testing machine (Instron model 2519-107, Instron, Norwood, MA, USA). Standard EN ISO 13934-1:2000 was applied to quantify the generated compression. The Laplace formula (Equation (1)) was employed to calculate the compression of the designed samples:

(1) $$P=\frac{2\cdot \pi \cdot F}{S}$$

where *p* = Pressure in Pa, *F* = Tensile force in N, *S* = Area of the sample in m^2^.

**Figure 2:**
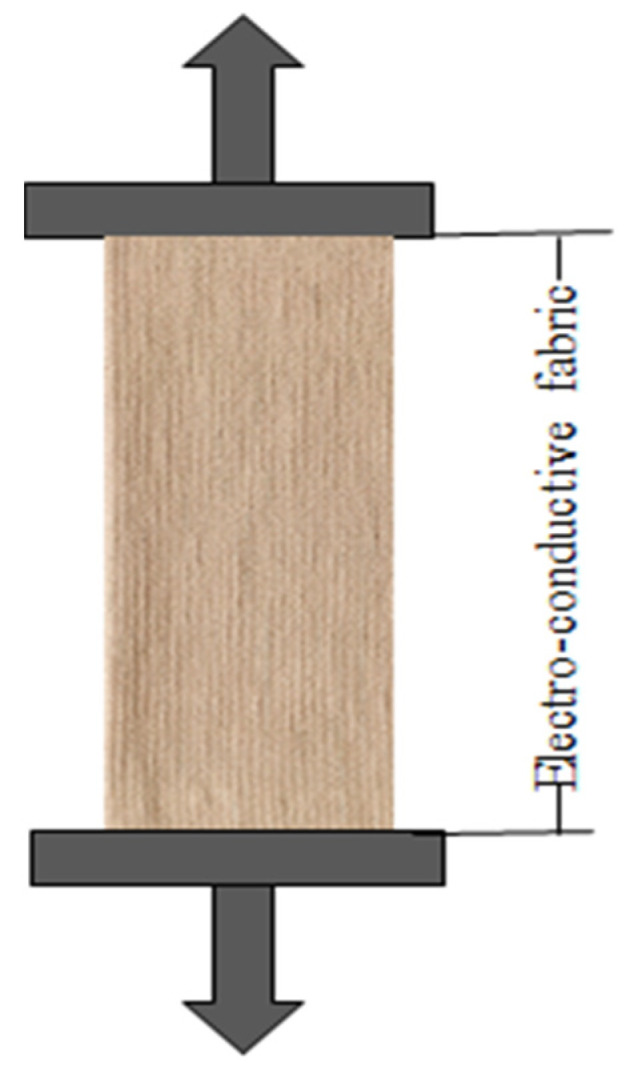
Experimental setup to measure the resistances and unidirectional tensile force.

**Figure 3:**
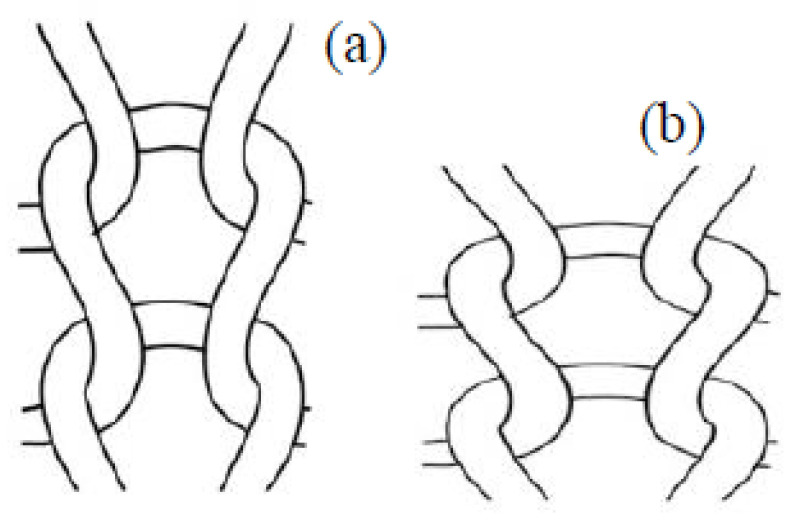
Loop changing presumption during stretching: (**a**) before stretching and (**b**) after stretching.

Two copper plates of specific dimensions were positioned on the two opposite sides of the composite fabric for measuring current dependence on voltage. The applied voltage was gradually increased, and ammeter readings of current across the structure were recorded. Figure 2 shows the experimental setup to measure the resistances and unidirectional tensile force. Figure 3 indicates the loop changing presumption during stretching.

**Figure 4:**
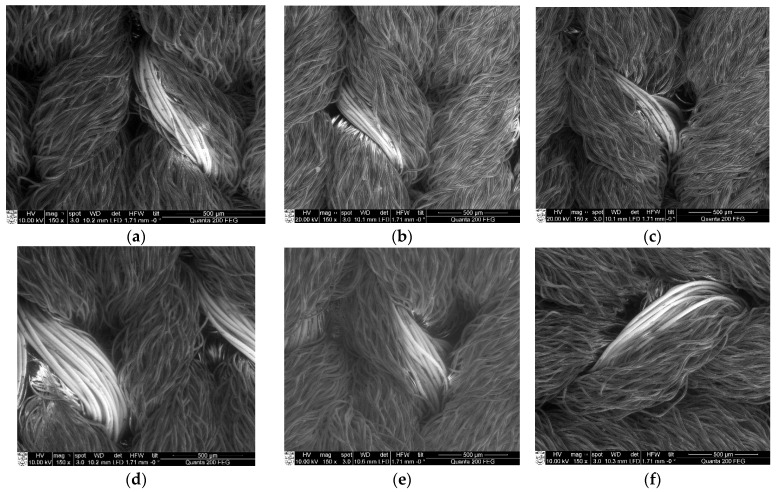
SEM illustrations of metal coated composite structure of (**a**) CF_LY1, (**b**) CF_LY2, (**c**) CF_LY3, (**d**) CF_HY1, (**e**) CF_HY2, and (**f**) CF_HY3 fabrics at 150× magnification.

**Figure 5:**
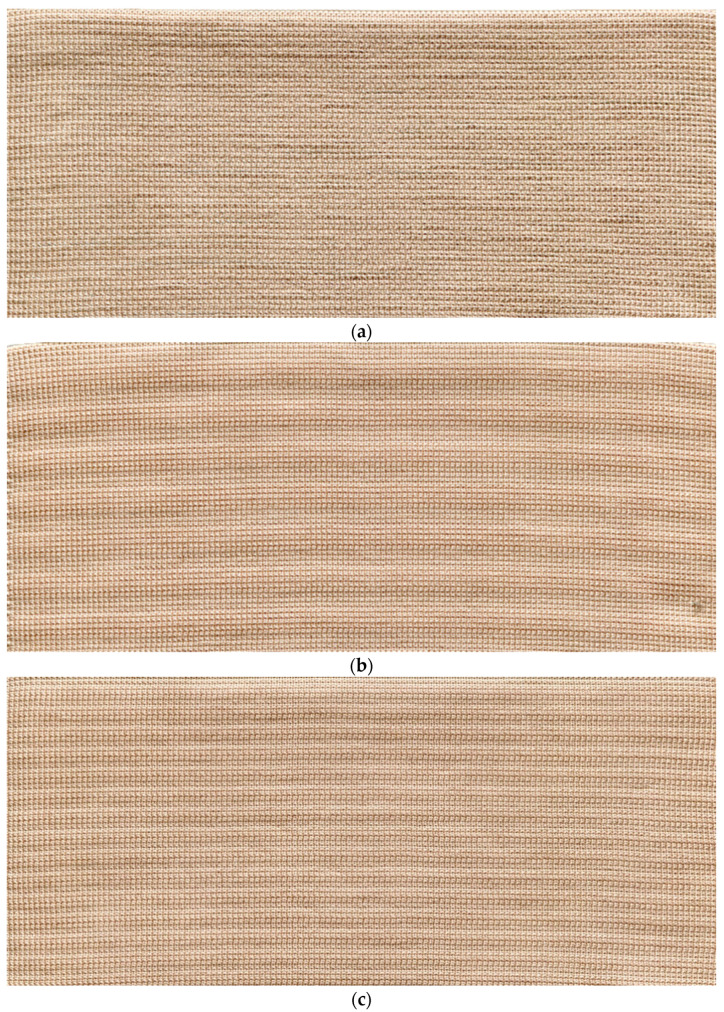
Snapshots of manufactured metal coated composite fabrics (left to right side indicates the course direction): (**a**) CF_LY1 and CF_HY1; (**b**) CF_LY2 and CF_HY2; and (**c**) CF_LY3 and CF_HY3.

In this research, similar elongation was chosen to generate required compression in the orthopaedic support and corresponding tensile forces were measured. At the same extension level, the stretching force is higher in the electro-conductive fabric of higher linear density yarn than in the lower linear density yarn. Figure 4 exhibits the SEM illustrations and Figure 5 displays the photographs of metal coated composite structures.

During the first minute of observation, the temperature was recorded every 10 s, and then every 20 s from the first minute to the end of the experiment.

All investigations were conducted in a controlled environment following LST EN ISO 139:2005.

## 3. Results and Discussion

### 3.1. Influence of Stretching on Temperature Behaviour Suring Voltage Applied

In order to find at what voltage what current is generated by each knitted specimen, it is important to reach the target temperature. Orthopaedic heated supports should be kept at a standard temperature of at least 40 °C to ensure that heating has a good effect on the healing process. Different voltage levels were selected, and performance was investigated. The unidirectional electrons flow source (DC power) was associated with the composite fabrics to measure current for corresponding voltage. The V–I scenario of fabricated composite samples are displayed in Table 2 and Table 3.

An electric current is transmitted across the conductive substances when electrodes are connected to a power source and heat is produced. Therefore, quantification of voltage–current (V–I) characteristics helps to forecast the heat generation manner of the conductive composite fabric. For orthopaedic supports, at least 40 °C temperature has to be reached. The presented data indicates that there is a minor effect of fabric stretching on current changes for the specific voltage applied. It can be also seen from the presented results that on the basis of voltage increasing levels, all designed composite samples show the linear current–voltage profile.

It was found that the current of the specimens of CF_LY1, CF_LY2 and CF_LY3 increases similarly by increasing voltage as the same linear density of yarn used in the composite fabric sample (see in Table 2). The matching scenario was observed for the specimens CF_HY1, CF_HY2 and CF_HY3 (see in Table 3). It is clearly evident from Table 2 and Table 3, that current generation decreases during increasing the stretching level at fixed applied voltage. This happens due to the pressed together of loops of conductive yarn during stretching and slightly impedes the flow of electrical current through fabric structure. Values of current of the specimens CF_LY1 and CF_HY1 at particular voltage are significantly higher if compared, accordingly, to the CF_LY2, CF_LY3, CF_HY2 and CF_HY3. This is due to the greater quantity of metal coated yarns in the structure and, respectively, less resistance. It was also confirmed that the current of the specimens CF_HY1, CF_HY2 and CF_HY3 were showed higher comparatively than CF_LY1, CF_LY2 and CF_LY3 as the linear density of yarn used was higher (see in Table 2 and Table 3). Moreover, this study disclosed the exciting tendency: there are some changes in current between composite structures with the comparatively same amount of the metal coated yarn in the structures, but different distribution of courses fabricated with metal coated yarn and without it, particularly at leading voltage. The results clearly demonstrate the importance of stretching, metal coated yarn linear density and knitting structures on the V–I characteristics. The higher the voltage, the higher the electric field in the conductive composite fabrics and the electric potential energy per charge transformed, as charges move in the designed area.

**Figure 6:**
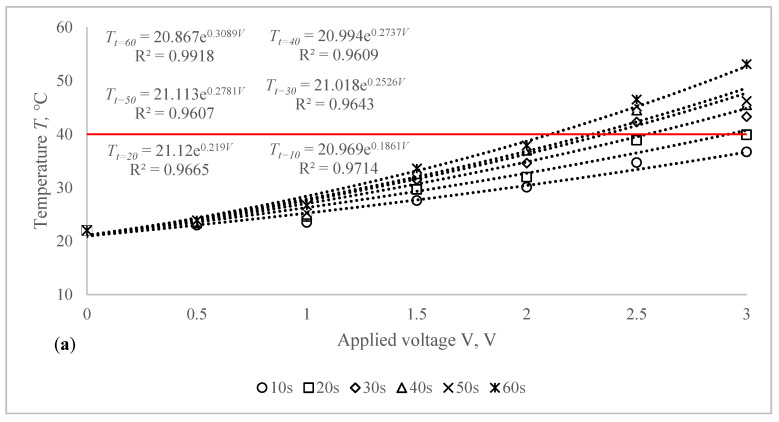

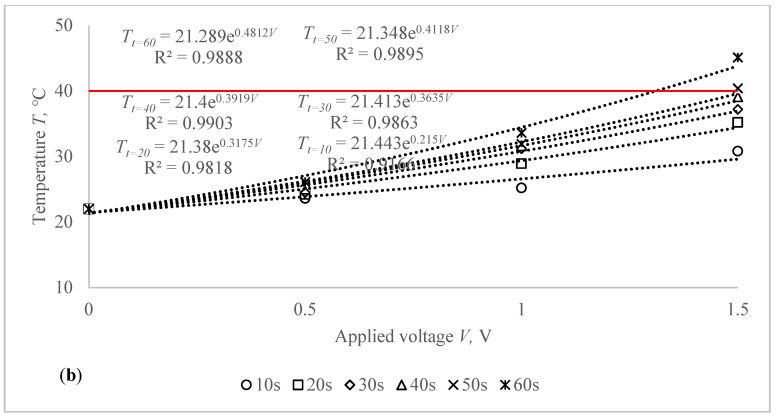
Voltage–temperature characteristics of non-stretched composite fabrics during 60 s at 10 s intervals for different applied voltage: (**a**) CF_LY1 and (**b**) CF_HY1 structured electro-conductive fabric (Red line indicates the target temperature).

The voltage–temperature (V–T) outlines of conductive composite fabrics were analysed using a variety of DC voltages. Temperature volumes on the composite samples were staged for 60 s. The results are presented in Figure 6 and Table 4. This behaviour was researched in order to demonstrate the heat generating behaviour over time. In all periods of observation, the dependence of the produced temperature on the applied voltage has an exponential nature (coefficient of determination R^2^ varies between 0.927 and 0.992).

The obtained results clearly indicate the influence of the time in temperature changes on the composite fabrics, especially at the higher values of the voltage applied. Additionally, the influence of the linear density of the metal coated yarn, stretching and fabric structure on the external temperature reached at the particular voltage is demonstrated in Figure 6 and Table 4. The higher voltage applied for the same resistance generates more current and it produces more energy, which is issued as heat and increases the temperature in the composite fabric surface. It is important that, at the higher voltage, the increase of temperature during the time is also significantly higher, especially for fabrics with higher resistance, in our case, for EF_LY group knits manufactured by utilizing 66 tex/12 filaments metal coated yarn.

By applying a voltage of 0.5 V, the temperature on the CF_LY1 composite fabric has increased during 50 s (from 10th till 60th s) in 1.0–1.9 °C, by applying 1 V voltage, the temperature has increased during the same time in 1.5–4.7 °C, by applying 1.5 V voltage, the temperature has increased in 5.6–11.6 °C, by applying 2 V voltage, the temperature has increased in 8.1–15.9 °C, by applying 2.5 V voltage, the temperature has increased in 12.7–24.5 °C and, by applying 3 V voltage, the temperature has increased during the same time in 14.7–31.1 °C at non-stretched state. For CF_LY2 structured conductive fabric, the increase of the temperature at 0.5 V voltage during 50 s was 0.8–1.7 °C degrees, while at 1 V voltage the temperature increased in 2.0–3.5 °C, at 1.5 V voltage the temperature increased in 4.4–6.7 °C, at 2 V voltage the temperature increased in 6.0–11.3 °C, at 2.5 V voltage the temperature increased in 6.4–15.5 °C, and, by applying 3 V voltage, the temperature increased during the same time in 14.1–25.5 °C degrees at non-stretched state. For CF_LY3 structured conductive fabric, the increase of the temperature at 0.5 V voltage during 50 s was 1.0–2.1 °C degrees, while at 1 V voltage the temperature increased in 1.7–3.3 °C, at 1.5 V voltage the temperature increased in 2.7–6.0 °C, at 2 V voltage the temperature increased in 3.9–9.7 °C, at 2.5 V voltage the temperature increased in 6.8–15.3 °C, and, by applying 3 V voltage, the temperature increased during the same time in 9.2–21.0 °C degrees at non-stretched state.

As shown in Table 4, stretching the compressive composite knitted fabric has a considerable negative influence on heat generation; nonetheless, the heat generation dynamics in both non-stretched and stretched states have a similar character during the voltage applied in constant time. The difference in temperature between non-stretched and 10% stretched states after 60 s was found to be 3.1 °C for CF_LY1, 2.5 °C for CF_LY2, and 0.8 °C for CF_LY3 when the applied voltage was 3 V, and this difference increases as the stretch level increases. It’s 4.3 °C, 4.2 °C, and 1.2 °C in the 15% stretch state, and 6.1 °C, 5.4 °C, and 2.0 °C in the 20% stretch state, respectively.

In EF_HY group knits knitted by using 235 tex conductive yarn, while applying a voltage of 0.5 V, the temperature on the surface of CF_HY1 structured conductive fabric increased during 50 s (from 10th till 60th s) in 1.6–4.1 °C degrees, by applying 1 V voltage, the temperature increased in 3.2–11.6 °C degrees and, by applying 1.5 V voltage, the temperature increased during the same time in 8.8–23.1 °C degrees at non-stretched state. For CF_HY2 conductive fabric, the increase of the temperature at 0.5 V voltage was 1.5–3.6 °C degrees, at 1 V voltage was 4.2–9.8 °C, and by applying 1.5 V voltage, the temperature increased during the same time in 9.7–17.8 °C degrees at non-stretched state. For CF_HY3 structured conductive fabric, the increase of the temperature at 0.5 V voltage during 50 s was 0.8–3.5 °C degrees, while at 1 V voltage the temperature increased in 3.2–8.6 °C and, by applying 1.5 V voltage, the temperature increased during the same time in 12.6–20.9 °C degrees at non-stretched state.

The stretch of the compressive composite knitted fabric also has a significant negative effect on the heat generation for EF_HY group structures, nevertheless, the heat generation dynamics throughout the voltage applied in constant time have a comparable character in both non-stretched and stretched states. Comparing temperatures in non-stretched and 10% stretched state, the difference after 60 s is 0.5 °C for CF_HY1, 0.7 °C for CF_HY2 and 2.4 °C for CF_HY3 while applied voltage was 1.5 V; in 15% state it is, accordingly, 1.7 °C, 2.4 °C and 3.3 °C; and in 20% state-accordingly, 3.6 °C, 3.7 °C and 4.4 °C. The results clearly demonstrate the importance of stretching on the V–T characteristics. The higher the voltage, the higher the electric field in the conductive surface and the heat generated.

**Figure 7:**
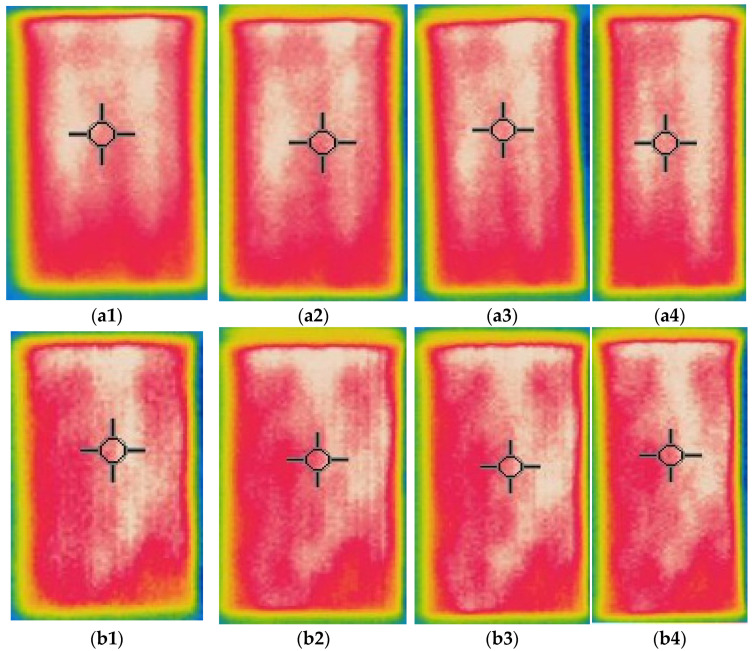

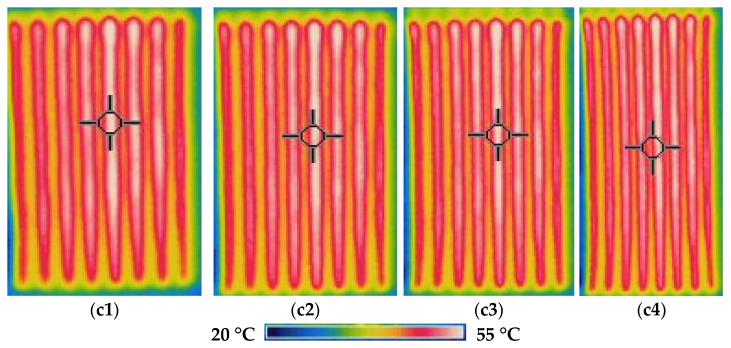
Thermal images of electro-conductive fabrics of CF_LY group after 600 s of 2.5 V application: infrared images at (**a1**) non-stretched, (**a2**) 10% stretched, (**a3**) 15% stretched and (**a4**) 20% stretched of CF_LY1 structured fabric; (**b1**) non-stretched, (**b2**) 10% stretched, (**b3**) 15% stretched and (**b4**) 20% stretched of CF_LY2 structured fabric; and (**c1**) non-stretched, (**c2**) 10% stretched, (**c3**) 15% stretched and (**c4**) 20% stretched of CF_LY3 structured fabric.

**Figure 8:**
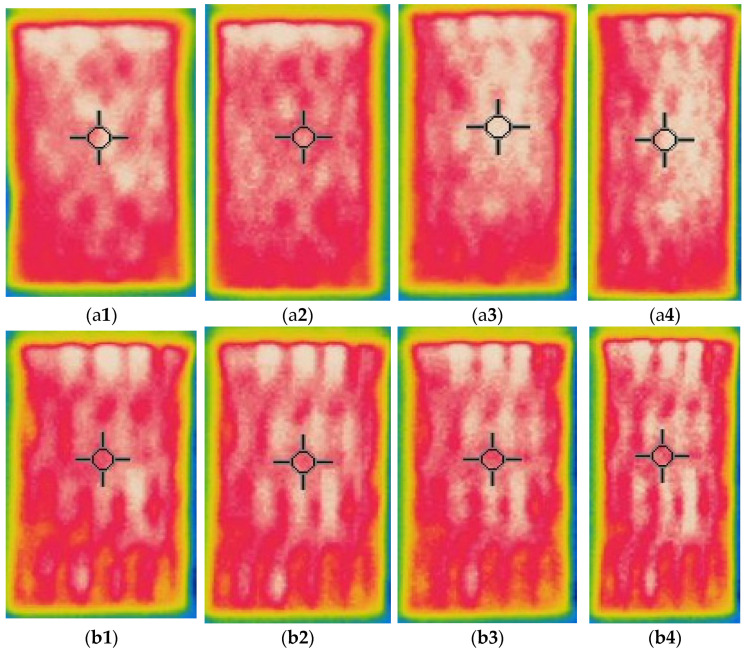

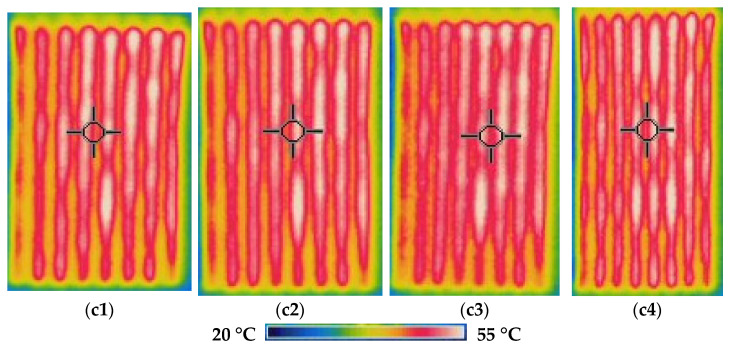
Thermal images of electro-conductive fabrics of CF_HY group after 600 s of 1.5 V application: infrared images at (**a1**) non-stretched, (**a2**) 10% stretched, (**a3**) 15% stretched and (**a4**) 20% stretched of CF_HY1 structured fabric; (**b1**) non-stretched, (**b2**) 10% stretched, (**b3**) 15% stretched and (**b4**) 20% stretched of CF_HY2 structured fabric; and (**c1**) non-stretched, (**c2**) 10% stretched, (**c3**) 15% stretched and (**c4**) 20% stretched of CF_HY3 structured fabric.

Thermal images of the composite fabrics between electrodes were captured after 600 s observations, by applying the constant 2.5 V voltage for the CF_LY group and by applying the constant 1.5 V voltage for the CF_HY group, images are presented in Figure 7 and Figure 8. The images were captured at 0–20% stretched condition.

The highest temperature is obtained on the surface of CF LY1 and CF HY1, which are knitted with metal coated yarns in each second course of the knit, as seen by thermal pictures (see in Figure 1). Due to the greater distance between courses with and without metal coated yarn, the temperature unevenness on the surface of the composite knitted constructions was shown for CF_LY2, CF_LY3, CF_HY2, and CF_HY3. Additionally, this unevenness is more pronounced in fabrics with lower conductive yarn linear density. The thermal images of CF_LY1 and CF HY1 show that these structures provide excellent evenness. The investigational results also revealed a temperature differential between the heating surface edges and the centre. The significant heat loss by radiation and air convection in the edge compared to the central position caused this scenario.

An electrical current flow through a structure when an electrical potential difference is applied on two entities of it or between two places. When a potential difference is applied across a mesh, the heating impact near the terminals is observed to be less, but the current density in the centre of the fabric is higher than at the edges, according to electrical principles. The effect creates non-uniform heating across the fabric’s surface [10]. To make the most exercise of the electro-conductive silver yarn, the knitted courses of silver yarn were separated using non-coated PA 6.6 ground yarn and elastomeric yarn in the current study.

It must be remembered that by introducing higher voltage (to achieve the desired temperature), the dynamic of temperature fluctuations over time is fairly slow, and this process must be studied over a longer period of time. As a result, in the following part of this research, temperature fluctuations on the surface of the freshly created knits were tested using different voltages for 600 s (10 min).

### 3.2. Influence of Stretching on Temperature Changes during the Time

Compression supports are elastic items that incorporate elastomeric yarns with an engineered compression gradient that can be worn on the limbs, upper, lower, or entire body for compression therapy. To provide the best compression generation, elastomeric inlay-yarns are employed in the knitted construction. Heated orthopaedic supports, too, require compression. As a result, the charge bearing yarns are bent into the loop rather than straight lay yarns in this arrangement. It permits for support extension to the necessary level without triggering damage to the metal coated conductive yarn. The specimens were stretched to a set elongation of 10%, 15%, and 20% in order to simulate reasonable wear circumstances and determine what effect this had on temperature generation. Table 5 shows the compression values obtained for the studied composite fabrics at various stretch levels.

**Figure 9:**
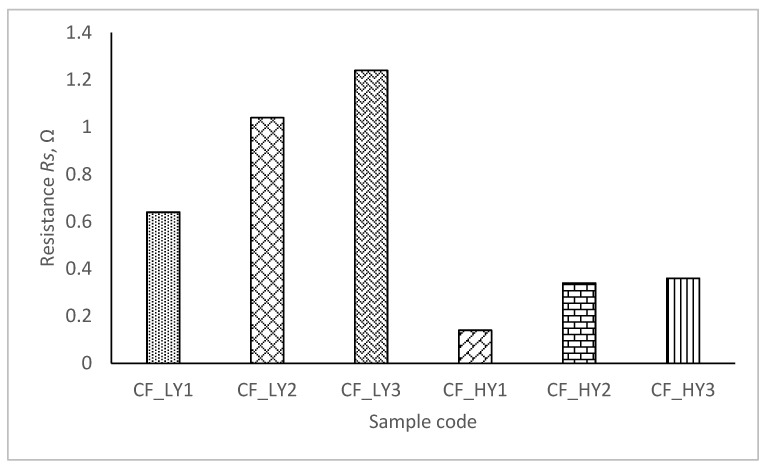
Resistance of CF_LY1, CF_LY2, CF_LY3, CF_HY1, CF_HY2 and CF_HY3 structured electro-conductive composite fabrics.

Figure 9 shows the resistance values of all manufactured composite specimens with varied layouts of the metal coated yarn in the structure.

The resistance values for all composite specimens are unique, as it is observed from the results reported in Figure 9. The quantity of metal coated yarn in the structure and the quantity of metal particles on the yarns have a big impact on the resistance of composite fabrics. With the reduction in the region of the conductive surface on the metal coated yarn in the knitting design, resistance rises steadily. The results show that the composite fabric’ resistance is influenced by the knitting structure, linear density of metal coated yarn, and amount of the metal coated yarn utilized in the knitting pattern. As a consequence of the higher linear density of the metal coated yarn and the higher number of filaments in the yarn, the CF_HY group of knits has lower resistance than the CF_LY group.

**Figure 10:**
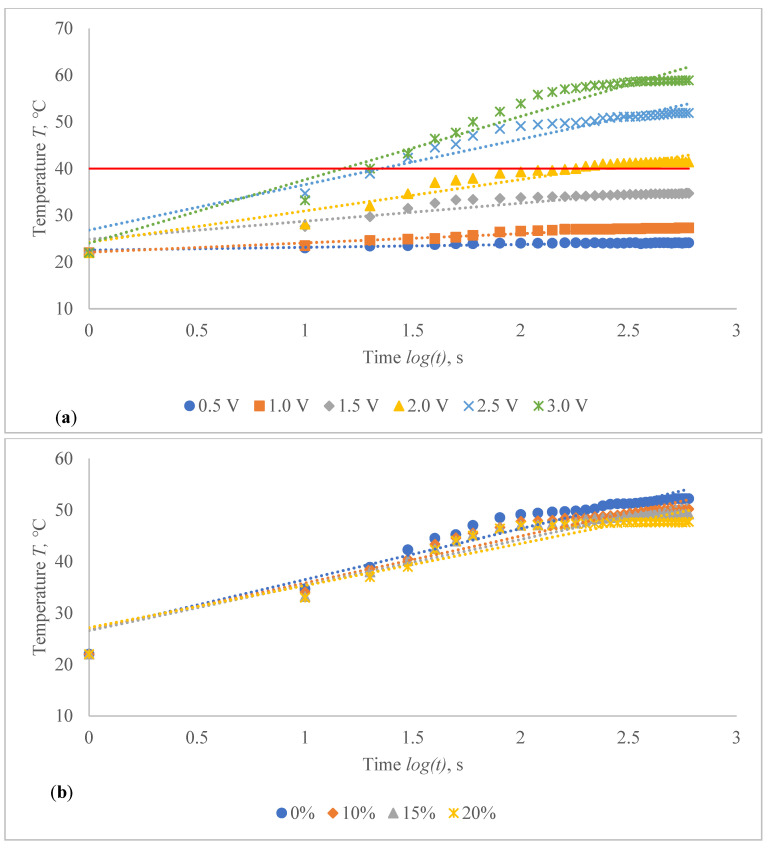
Temperature changes of CF_LY1 structured electro-conductive composite fabric during the 600 s period: (**a**) at non-stretched state by applying different voltage; and (**b**) at the different stretch levels by applying 2.5 V. Red line indicates the target temperature.

**Figure 11:**
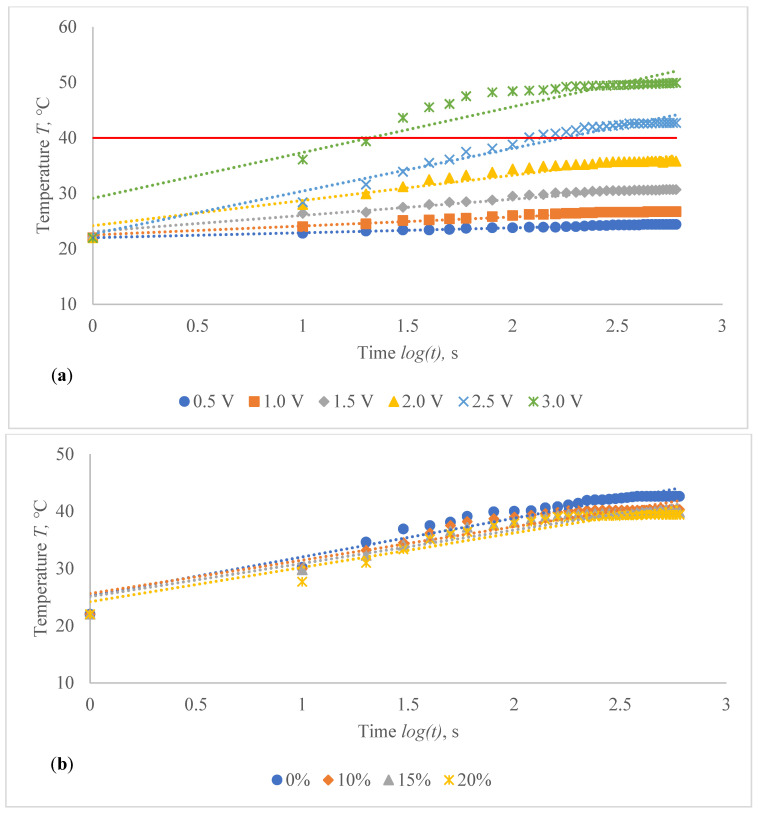
Temperature changes of CF_LY2 structured electro-conductive composite fabric during the 600 s period: (**a**) at non-stretched state by applying different voltage; and (**b**) at the different stretch levels by applying 2.5 V. Red line indicates the target temperature.

**Figure 12:**
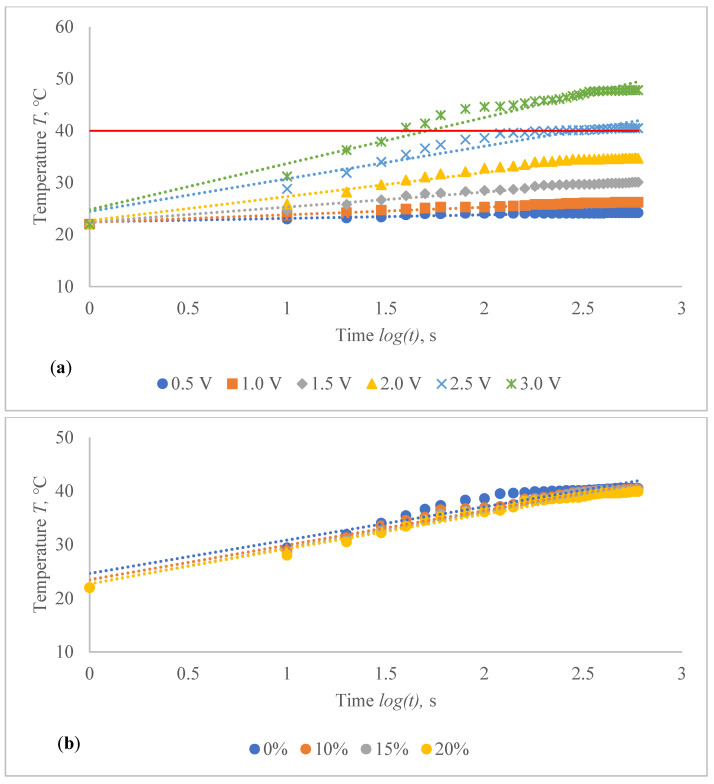
Temperature changes of CF_LY3 structured electro-conductive composite fabric during the 600 s period: (**a**) at non-stretched state by applying different voltage; and (**b**) at the different stretch levels by applying 2.5 V. Red line indicates the target temperature.

**Figure 13:**
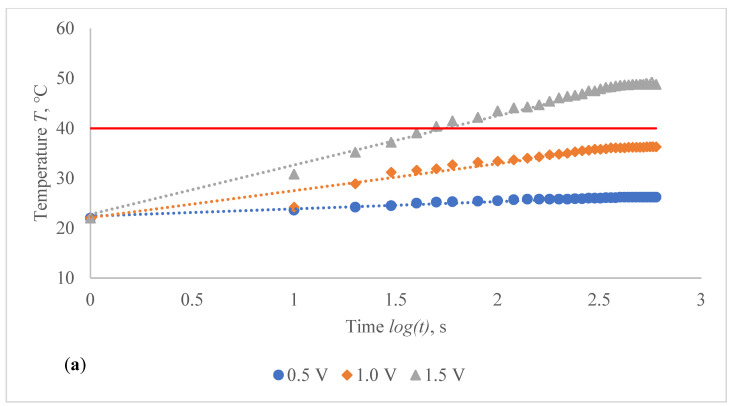

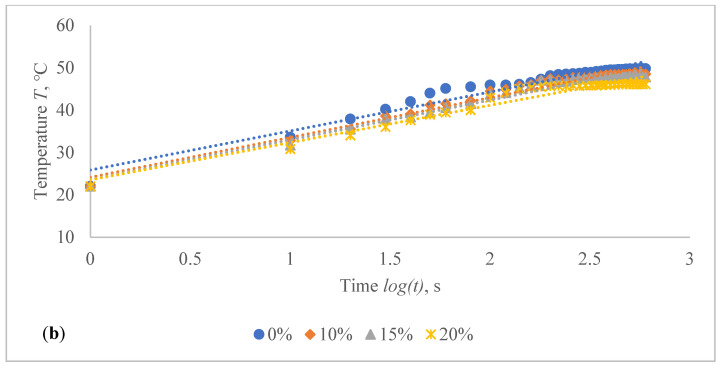
Temperature changes of CF_HY1 structured electro-conductive composite fabric during the 600 s period: (**a**) at non-stretched state by applying different voltage; and (**b**) at the different stretch levels by applying 1.5 V. Red line indicates the target temperature.

**Figure 14:**
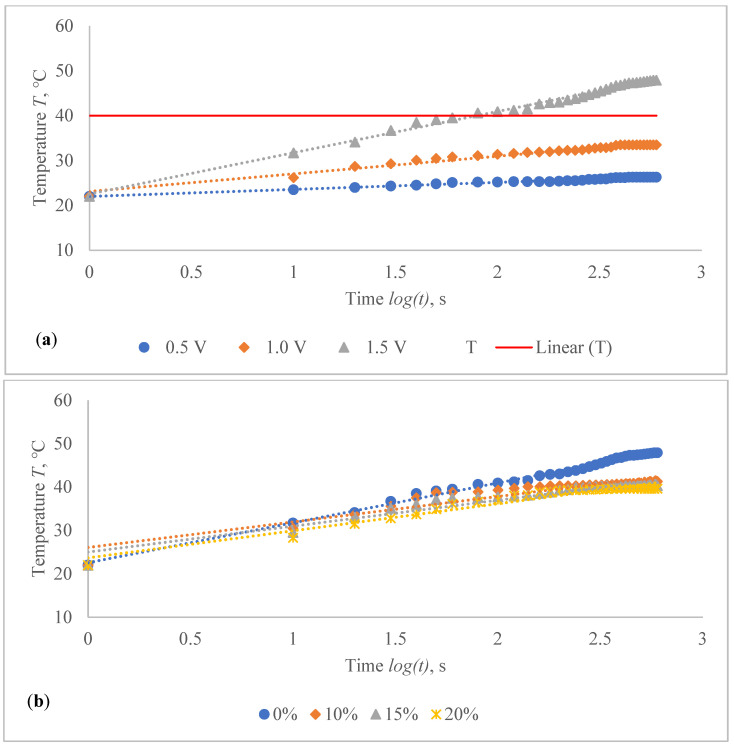
Temperature changes of CF_HY2 structured electro-conductive composite fabric during the 600 s period: (**a**) at non-stretched state by applying different voltage; and (**b**) at the different stretch levels by applying 1.5 V. Red line indicates the target temperature.

**Figure 15:**
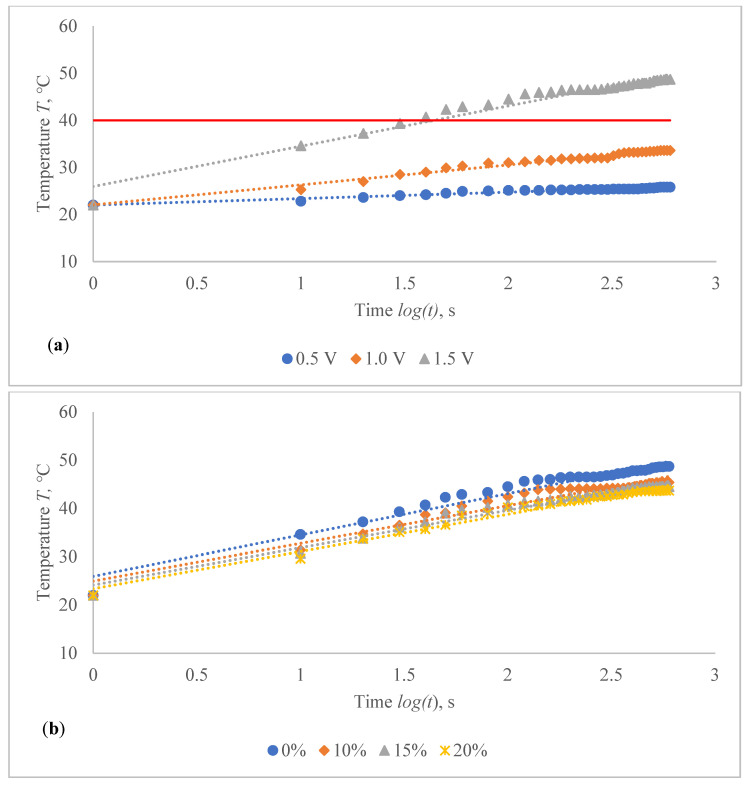
Temperature changes of CF_HY3 structured electro-conductive composite fabric during the 600 s period: (**a**) at non-stretched state by applying different voltage; and (**b**) at the different stretch levels by applying 1.5 V. Red line indicates the target temperature.

The temperature profile of developed highly sensitive Ag coated composite fabric was explored, and the achieved findings are shown in Figure 10, Figure 11, Figure 12, Figure 13, Figure 14 and Figure 15. The surface of the composite fabric was exposed to several DC voltage levels such as 0.5, 1.0, 1.5, 2.0, 2.5 and 3.0 V. Temperature of the composite fabric was then monitored at a set voltage level after every 10 s during the first minute, and after every 20 s from the first minute until the end of the assessment, i.e., until 10 min (600 s). In all voltages applied to observation, the dependence of the temperature on time has a logarithmic character (coefficients of determination R^2^ vary between 0.826 and 0.986).

The results indicated in Figure 10, Figure 11, Figure 12, Figure 13, Figure 14 and Figure 15 show that the temperature rises quickly at the beginning, but after 2 min, the temperature rise extremely slows down for all voltages applied until it achieves a constant value. For heat therapy, maintenance of a consistent temperature for an extended period of time is particularly effective. The temperature behaviour is reasonable: as the temperature rises, the heat lost through radiation and air convection rises as well, eventually achieving a balance between heat created by current and heat lost. Due to the higher linear density of the metal coated yarn, the temperature rises for the CF_HY substantially more than for the CF_LY. As a result, it is safe to assume that this electro-conductive composite fabric will heat up quickly and level off at a specific temperature. This property makes it ideal for use as a heating component in orthopaedic supports. The applied voltage and time are optimized to provide a suitable temperature on human skin, produced by the textile heating structure based on numerous criteria. The applied voltage of 2.5 V is enough for the CF_LY group to reach the target temperature and 1.5 V for the CF_HY group.

The temperature on the CF_LY1 structured composite fabric was increased in 1.0–2.1 °C from 10th to 600th s while applying 0.5 V voltage, 1.5–5.3 °C at 1.0 V, 5.6–12.7 °C at 1.5 V, 6.1–19.4 °C at 2.0 V, 12.7–29.9 °C at 2.5 V, and 11.2–36.9 °C by applying 3 V voltage at non-stretched state (see in Figure 10a). The temperature of the CF_LY2 structured composite fabric was increased in 0.8–2.4 °C while applying 0.5 V voltage from 10th to 600th s; at 1.0 V voltage, the temperature increased in 2.0–4.7 °C; at 1.5 V voltage, the temperature increased in 4.4–8.7 °C; at 2.0 V voltage, the temperature increased in 6.0–13.8 °C; at 2.5 V voltage, the temperature increased in 6.4–20.7 °C; and at 3.0 V voltage, the temperature increased in 14.1–27.9 °C (see in Figure 11a). For CF_LY3 structured composite fabric, the increase of the temperature at 0.5 V voltage during 10th till 600th s was 1.0–2.2 °C, while applying 1.0 V voltage the temperature increased in 1.7–4.2 °C, at 1.5 V voltage the temperature increased in 2.7–8.1 °C, at 2.0 V voltage the temperature increased in 2.9–12.7 °C, at 2.5 V voltage the temperature increased in 6.8–18.5 °C, and, by applying 3.0 V voltage, the temperature increased during the same time in 9.2–25.8 °C degrees at non-stretched state (see in Figure 12a).

The stretch of the compressive composite fabric has a considerable negative influence on heat generation, as shown in Figure 10, Figure 11 and Figure 12; however, the heat generation dynamics during the time at constant voltage are identical in both non-stretched and stretched states. The difference in temperature between non-stretched and 10% stretched states after 600 s was found to be 2.0 °C for CF_LY1, 2.3 °C for CF_LY2, and 0.2 °C for CF_LY3 when the applied voltage was 2.5 V, and this difference increases as the stretch level increases. It’s 3.0 °C, 2.8 °C, and 0.3 °C in the 15% stretch state, and 4.5 °C, 3.2 °C, and 0.6 °C in the 20% stretch state, respectively.

While applying 0.5 V voltage on the CF_HY1 structured composite fabric, the temperature increased in 1.6–4.2 °C during 10th to 600th s, when applying 1.0 V voltage, the temperature increased in 2.2–14.3 °C, and when applying 1.5 V voltage, the temperature increased in 8.8–26.8 °C during the same time at non-stretched state in CF_HY group knits fabricated with 235 tex/34 filaments metal coated yarn (see in Figure 13a). For CF_HY2 conductive fabric, the increase of the temperature at 0.5 V voltage was 1.5–4.3 °C degrees during 10th s till 600th s, at 1 V voltage was 4.2–11.5 °C, and, by applying 1.5 V voltage, the temperature increased during the same time in 9.7–25.9 °C degrees at non-stretched state (see in Figure 14a). For CF_HY3structured conductive fabric, the increase of the temperature at 0.5 V voltage during 10th till 600th s was 0.8–3.8 °C degrees, while at 1 V voltage the temperature increased in 3.2–11.6 °C and, by applying 1.5 V voltage, the temperature increased during the same time in 12.6–26.7 °C degrees at non-stretched state (see in Figure 15a).

For CF_HY group structures, the stretch of the compressive composite knitted structure has a considerably negative effect on heat generation; nonetheless, the heat generation dynamics during the voltage applied in constant time are identical in both non-stretched and stretched states. When comparing temperatures in non-stretched and 10% stretched states after 600 s, the difference is 0.3 °C for CF_HY1, 6.6 °C for CF_HY2, and 3.3 °C for CF_HY3 when applied voltage was 1.5 V; in 15% state, it is 1.0 °C, 7.6 °C, and 4.2 °C; and in 20% state, it is 1.3 °C, 8.1 °C, and 5.0 °C. The findings show that stretching has a significant impact on *t-T* properties.

The collected results reveal that the target temperature may not be reached when applying for compression support in the stretched state required to achieve some specific compression. The decrease in temperature with stretch is due to an increase in surface area of the composite structure during stretching, while the amount of metal coated yarn in the sample continues steadily. The tensile force contributes to the displacement of the contact point by counteracting the friction force that occurs at the contact point between the loops during the stretch of the composite fabric. The bending curve of the yarn is also changed. Additionally, as the contact points alter, the length of the limbs, head, and sinker loop shifts. As the tensile force increases, the degree of movement of the contact points decreases, and the contact pressure between the head and sinker loop rapidly rises. The resistance of the composite fabric along the course direction might be elucidated by superimposing the length-related and contact resistances. The contact resistance decreases during the early stretching phase, whereas the length-related resistance dominates the total equivalent resistance in the stretching process for future tensile force rise [22,23,24]. Due to frictional abrasion, the contact resistance should decrease as the contact force increases in metal doped polymers [25]. However, it was discovered in this study that the electrical resistance can be maintained with only a small amount of contact force. Unless the length or cross sectional area of the contacting points changes, the contact resistance will not vary. As demonstrated in Figure 9, Figure 10, Figure 11, Figure 12, Figure 13 and Figure 14, as the proportion of strain increases, the temperature of the heating element lowers significantly. The cause for the drop in temperature was not related to a change in the electrical resistance of the heating element, but rather to the heating element’s surface area becoming porous [10]. As the area of designed composite fabrics rises during stretching, the amount of heat lost through radiation and air convection increases. Finally, the heat saturation is reached between heat creation and heat loss [4]. As a result, while designing heated compression supports, the stretch effect on heat generation must be considered.

All of the designed and examined structures can be utilized for compressive orthopaedic heated supports, however, the higher linear density of the metal coated yarn necessitates a lower energy source to obtain the desired temperature, which is an advantage. Furthermore, as compared to equivalent constructions knitted with lower linear density metal coated yarn, the unevenness of temperature on the surface of composite fabrics of CF_HY2 and CF_HY3 is substantially reduced. It means that the amount of expensive Ag coated yarn used in the knitted construction can be cut in half without compromising the heating capabilities.

## 4. Conclusions

The presented research revealed prospects to use knitted composite textile structure with inserted metal coated yarn for heat generation in orthopaedic compression supports. To secure the structure from mechanical damage, silver coated polyamide yarns were incorporated into a combined half-Milano rib structure plated by the PA6.6 yarn. Temperature of the heated samples rises rapidly for the first minute, after which the growth gradually decreases and eventually stabilizes. Depending on the structure and voltage applied, the generally stable 40 °C heating temperature was obtained in about 2–3 min. It was found that due to the increase of the surface area, the stretch of the specimen has a negative impact on heat generation. The designed composite structure developed in this study can be utilized to provide suitable heat to the orthopaedic compression supports; however, the higher linear density of the metal coated yarn requires a lower voltage energy source to provide the target 40 °C. For CF_LY group specimens, 2.5 V voltage is enough to reach the target temperature, while 1.5 V is needed for CF_HY group specimens. After 600 s heating period of CF_LY variants, the temperature decreased by approximately 4.5 °C for CF_LY1, 3.2 °C for CF_LY2 and 0.6 °C for CF_LY3 while applied voltage was 2.5 V at 20% stretch. For CF_HY variants, the temperature decreased by approximately 3.7 °C for CF_HY1, 8.3 °C for CF_HY2 and 5.0 °C for CF_HY3 while applied voltage was 1.5 V at the same stretch level. The identical trend was detected at all stretch levels. The stretch has an impact on both the temperature and the time it takes to reach the desired temperature. The stretch has a slightly less impact on the CF_LY group fabricated with silver coated PA yarns of 66 tex/12 filaments than it does on the CF_HY group fabricated with silver coated PA yarns of 235 tex/34 filaments. Thereupon, the effect of stretch on heat generation reduction must be considered while designing a new compression heated support.

To boost the effectiveness of knit compression garments for medical products, the authors will investigate the possible outcomes of thermoregulation due to structural and morphological changes under cyclic deformation and washing impact (more than 20 wash) in the next research stage.

## Figures and Tables

**Table 1:** Essential parameters of designed composite fabrics.

Sample Code	Area Density, g/m^2^	Wale *P_w_* and Course *P_c_* Density (cm^−1^)				Average Loop Length, (mm)	Sample Area, m^2^
		Technical Face Side		Technical Back Side			
		*P_c_*	*P_w_*	*P_c_*	*P_w_*		
CF_LY1	326 ± 2	6.0 ± 0.1	6.0 ± 0.1	12.0 ± 0.1	6.0 ± 0.1	8.2 ± 0.3	0.022
CF_LY2	324 ± 2	6.0 ± 0.1	6.0 ± 0.1	12.0 ± 0.1	6.0 ± 0.1	8.2 ± 0.2	0.022
CF_LY3	325 ± 2	6.0 ± 0.1	6.0 ± 0.1	12.0 ± 0.1	6.0 ± 0.1	8.2 ± 0.3	0.022
CF_HY1	351 ± 2	6.0 ± 0.1	6.0 ± 0.1	12.0 ± 0.1	6.0 ± 0.1	8.1 ± 0.2	0.022
CF_HY2	349 ± 2	6.0 ± 0.1	6.0 ± 0.1	12.0 ± 0.1	6.0 ± 0.1	8.1 ± 0.2	0.022
CF_HY3	350 ± 2	6.0 ± 0.1	6.0 ± 0.1	12.0 ± 0.1	6.0 ± 0.1	8.1 ± 0.3	0.022

**Table 2:** Influence of stretching on the current *I* generated in CF_LY1, CF_LY2 and CF_LY3 during employing different voltage V.

Sample Code	Stretch, %	Current I, A					
		0.5 V	1.0 V	1.5 V	2.0 V	2.5 V	3.0 V
CF_LY1	0	0.76	1.52	2.26	3.02	3.73	4.49
	10	0.75	1.50	2.25	2.99	3.71	4.46
	15	0.75	1.48	2.21	2.86	3.59	4.39
	20	0.69	1.43	2.12	2.80	3.54	4.29
CF_LY2	0	0.49	0.98	1.45	1.92	2.38	2.81
	10	0.48	0.96	1.42	1.88	2.35	2.76
	15	0.47	0.95	1.42	1.85	2.32	2.74
	20	0.46	0.95	1.41	1.84	2.3	2.72
CF_LY3	0	0.45	0.91	1.35	1.74	2.15	2.55
	10	0.44	0.88	1.33	1.73	2.14	2.53
	15	0.44	0.86	1.31	1.73	2.13	2.53
	20	0.42	0.82	1.28	1.71	2.10	2.50

**Table 3:** Influence of stretching on the current I generated in CF_HY1, CF_HY2 and CF_HY3 during employing different voltage V.

Sample Code	Stretch, %	Current I, A		
		0.5 V	1.0 V	1.5 V
CF_HY1	0	2.89	5.93	8.92
	10	2.87	5.8	8.81
	15	2.57	5.74	8.6
	20	2.53	5.65	8.41
CF_HY2	0	1.67	3.52	5.57
	10	1.64	3.26	4.91
	15	1.6	3.16	4.76
	20	1.48	3.04	4.58
CF_HY3	0	1.64	3.48	6.59
	10	1.57	3.16	4.83
	15	1.57	3.15	4.72
	20	1.51	3.13	4.67

**Table 4:** Voltage–temperature characteristics at different stretching levels of developed electro-conductive composite fabrics for 60 s.

Sample Code	Applied Voltage *V*, V	Temperature *T*, °C			
		Stretch, 0%	Stretch, 10%	Stretch, 15%	Stretch, 20%
CF_LY1	0.5	23.9	23.5	23.5	23.3
	1.0	26.7	26.2	26	26
	1.5	33.6	31.5	30.8	30
	2.0	37.9	37.4	37	36.7
	2.5	46.5	44.5	43.7	42.9
	3.0	53.1	50	48.8	47
CF_LY2	0.5	24.2	23.8	23.9	23.7
	1.0	26.1	25.6	25.5	25.6
	1.5	29.8	29	28.5	28.2
	2.0	33.3	32.7	32.5	31.3
	2.5	39.5	38.2	37.2	36.4
	3.0	47.5	45	43.3	42.1
CF_LY3	0.5	24.1	23.7	23.6	22.9
	1.0	25.3	25	25	24.6
	1.5	28	27.6	27.9	27.3
	2.0	31.7	31.4	31.2	30.4
	2.5	37.3	36.5	35.7	35.3
	3.0	43	42.2	41.8	41
CF_HY1	0.5	26.1	25.6	25.3	24.8
	1.0	33.6	32.7	32.3	30.7
	1.5	45.1	44.6	43.4	41.5
CF_HY2	0.5	25.6	25.4	25	24.4
	1.0	31.8	29.8	29.5	29.3
	1.5	39.8	39.1	37.4	36.1
CF_HY3	0.5	25.5	24.8	24.7	24.7
	1.0	30.9	30.1	29.6	29.1
	1.5	42.9	40.5	39.6	38.5

**Table 5:** Tensile force and corresponding compression at the different stretch levels.

Sample Code	Amount of Load *F*, N for Stretching			Amount of Generated Compression *p*, kPa		
	10%	15%	20%	10%	15%	20%
CF_LY1	5.00	6.12	7.37	1.43	1.75	2.10
CF_LY2	5.29	6.75	8.03	1.51	1.93	2.29
CF_LY3	5.50	7.02	8.40	1.57	2.01	2.39
CF_HY1	5.26	6.91	8.15	1.50	1.97	2.33
CF_HY2	5.38	6.90	8.31	1.54	1.97	2.37
CF_HY3	5.24	7.06	8.19	1.49	2.02	2.34

## Data Availability

Not applicable.
